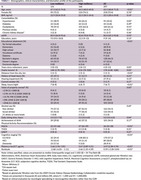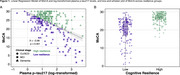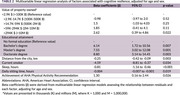# The Effect of Social Determinants of Health on Cognitive Resilience to Alzheimer's Disease, Determined by Plasma *p*‐tau217 in the Prospective INDE Cohort in Thailand: A Story from a Middle‐Income Country

**DOI:** 10.1002/alz70860_106910

**Published:** 2025-12-23

**Authors:** Thanapoom Taweephol, Thanakit Pongpitakmetha, Akarin Hiransuthikul, Kittithatch Booncharoen, Watayuth Luechaipanit, Thanaporn Haethaisong, Adipa Chongsuksantikul, Prawit Oangkhana, Poosanu Thanapornsangsuth

**Affiliations:** ^1^ Department of Microbiology, Faculty of Medicine, Chulalongkorn University, Bangkok, Thailand; ^2^ Department of Pharmacology, Faculty of Medicine, Chulalongkorn University, Bangkok, Thailand; ^3^ Division of Neurology, Department of Medicine, Faculty of Medicine, Chulalongkorn University, Bangkok, Thailand; ^4^ Chula Neuroscience Center, King Chulalongkorn Memorial Hospital, The Thai Red Cross Society, Bangkok, Thailand; ^5^ Memory Clinic, King Chulalongkorn Memorial Hospital, The Thai Red Cross Society, Bangkok, Thailand; ^6^ Department of Preventive and Social Medicine, Faculty of Medicine, Chulalongkorn University, Bangkok, Thailand; ^7^ Neurology Center, Phyathai 1 Hospital, Bangkok, Rachathewi, Thailand; ^8^ Thai Red Cross Emerging Infectious Diseases Health Science Centre, World Health Organization Collaborating Centre for Research and Training on Viral Zoonoses, King Chulalongkorn Memorial Hospital, The Thai Red Cross Society, Bangkok, Thailand; ^9^ Thai Red Cross Emerging Infectious Diseases Health Science Centre, King Chulalongkorn Memorial Hospital, The Thai Red Cross Society, Bangkok, Thailand

## Abstract

**Background:**

Social determinants of health (SDOH) contribute to cognitive resilience, ranging from healthy populations to aging individuals with cognitive impairment, including those with Alzheimer's disease (AD) and related dementias. Plasma phosphorylated tau 217 (*p*‐tau217) has been proven to be a specific biomarker reflecting AD pathology and severity (Jack et al., 2024). We aim to assess factors contributing to cognitive resilience among the Thai population.

**Method:**

We prospectively enrolled participants into the INDE cohort in King Chulalongkorn Memorial Hospital, Bangkok, Thailand (NCT06375213), collecting exhaustive clinical information, neuropsychological tests, and plasma *p*‐tau217. A regression model was fitted with Montreal Cognitive Assessment (MoCA) scores and plasma *p*‐tau217 levels to calculate residuals, representing cognitive resilience. Positive residuals indicate high resilience, whereas negative residuals indicate low resilience. A second regression model examined factors associated with cognitive resilience, focusing on each SDOH, with adjustments for age and sex.

**Result:**

Among 297 participants (73.4% female and median age 66 years [IQR: 61, 71]), 166 (55.9%) had high resilience (Table 1). A linear regression model showed an inverse relationship between MoCA and log‐transformed plasma *p*‐tau217 levels (β = ‐3.96, *p* < 0.01), and a box‐and‐whisker plot illustrated MoCA distribution across resilience groups (Figure 1). Multivariable linear regression analysis demonstrated that property ownership exceeding 10‐million‐baht, higher educational attainment, and meeting American Heart Association (AHA) physical activity recommendations were significantly associated with greater cognitive resilience. Higher education level correlated with increased resilience in a progressive manner. In contrast, greater distance from the city, current smoking, longer sleep duration, and increased daily sitting time were significantly linked to lower resilience (Table 2).

**Conclusion:**

SDOH notably impacted cognitive resilience, determined by the residuals of *p*‐tau217 and cognitive score. Public policy and clinical intervention regarding factors associated with cognitive resilience are warranted, even in low‐ and middle‐income countries (LMICs).